# Retraction Note: β-Trcp and CK1δ-mediated degradation of LZTS2 activates PI3K/AKT signaling to drive tumorigenesis and metastasis in hepatocellular carcinoma

**DOI:** 10.1038/s41388-025-03344-w

**Published:** 2025-03-17

**Authors:** Yanwei Lu, Xudong Li, Hongli Liu, Jun Xue, Zhen Zeng, Xiaorong Dong, Tao Zhang, Gang Wu, Kunyu Yang, Shuangbing Xu

**Affiliations:** Cancer Center, Union Hospital, Tongji Medical College, Huazhong University of Science and Technology, Wuhan, 430022 China

Retraction to: *Oncogene* 10.1038/s41388-020-01596-2, published online 08 January 2021

The Editors-in-Chief have retracted this article. After publication, the authors found that three images in Figs. 3d and 4g had been misused, and that the SMMC-7721 and L-02 cell lines used in the in vitro experiments were contaminated with HeLa cervical cancer cells.

The Editors-in-Chief therefore no longer have confidence in the presented data.

Yanwei Lu and Shuangbing Xu disagree with this retraction. None of the other authors have responded to correspondence from the Publisher about this retraction.

